# The Protein-Binding Behavior of Platinum Anticancer Drugs in Blood Revealed by Mass Spectrometry

**DOI:** 10.3390/ph14020104

**Published:** 2021-01-29

**Authors:** Jingchen Wang, Jianmei Tao, Shuailong Jia, Meiqin Wang, Hongliang Jiang, Zhifeng Du

**Affiliations:** School of Pharmacy, Huazhong University of Science and Technology, Wuhan 430030, China; m202075585@hust.edu.cn (J.W.); m202075554@hust.edu.cn (J.T.); d201881324@hust.edu.cn (S.J.); wangmeiqin@hust.edu.cn (M.W.); jianghongliang@hust.edu.cn (H.J.)

**Keywords:** platinum, anticancer drugs, blood, binding proteins, mass spectrometry

## Abstract

Cisplatin and its analogues are widely used as chemotherapeutic agents in clinical practice. After being intravenously administrated, a substantial amount of platinum will bind with proteins in the blood. This binding is vital for the transport, distribution, and metabolism of drugs; however, toxicity can also occur from the irreversible binding between biologically active proteins and platinum drugs. Therefore, it is very important to study the protein-binding behavior of platinum drugs in blood. This review summarizes mass spectrometry-based strategies to identify and quantitate the proteins binding with platinum anticancer drugs in blood, such as offline high-performance liquid chromatography/inductively coupled plasma mass spectrometry (HPLC–ICP-MS) combined with electrospray ionization mass spectrometry (ESI-MS/MS) and multidimensional LC–ESI-MS/MS. The identification of in vivo targets in blood cannot be accomplished without first studying the protein-binding behavior of platinum drugs in vitro; therefore, relevant studies are also summarized. This knowledge will further our understanding of the pharmacokinetics and toxicity of platinum anticancer drugs, and it will be beneficial for the rational design of metal-based anticancer drugs.

## 1. Introduction

**Figure 1:**
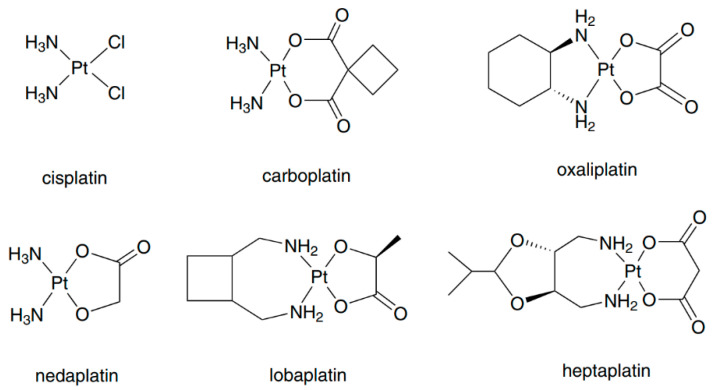
Structure of clinically used platinum anticancer drugs. Reprinted with permission from [6]. Copyright Springer 2016.

Cisplatin (Figure 1), which was discovered by Rosenberg in the 1960s, is the first metal complex to exhibit antitumor activity [1]. Clinical studies have shown that cisplatin can be used for the treatment of various cancers [2]; however, it also has severe side-effects such as renal damage, bone marrow suppression, and peripheral neuropathy [2,3]. Thereafter, second-generation platinum drugs carboplatin, nedaplatin and third-generation platinum drugs oxaliplatin, lobaplatin and heptaplatin (Figure 1) were developed to reduce the side-effects and increase the antitumor spectrum [4,5]. Except these clinically used platinum(II) compounds, other types of platinum compounds including polynuclear platinum complexes and platinum(IV) prodrugs are potential drug candidates [6]. Platinum(II) drugs are among the most widely used anticancer drugs. Among them, carboplatin can be used for the treatment of ovarian cancer and non-small-cell lung cancer with fewer side effects [7]. The main form of toxicity due to carboplatin is myelosuppression [8]. Oxaliplatin was reported to be active against cisplatin-resistant cell lines and it was found to have reversible neurotoxicity [9,10,11]. Nedaplatin shows similar cytotoxicity to but less nephrotoxicity than cisplatin [12], and it can be used for the treatment of genitourinary, head, and neck cancers [13,14,15]. Myelosuppression is also the main form of toxicity due to nedaplatin [16].

The pharmacokinetics of platinum anticancer drugs have been extensively studied. The differences among them were attributed to the different leaving groups. The formulation and protein-binding behavior of platinum drugs in plasma have a great impact on their pharmacokinetics, for example renal excretion rate [6,17,18]. Generally, if the leaving group of platinum complexes cannot be easily replaced by a ligand, their protein-binding ratio will be lower, their half-life will be longer, and their rate of renal excretion will be higher. The pharmacokinetic data for cisplatin, carboplatin, and oxaliplatin are summarized in Table 1 according to the literature [19].

After intravenous infusion, cisplatin rapidly diffuses into tissues, and more than 90% binds to plasma protein [20]. More specifically, 24 h after infusion, the protein-binding rate (PBR) for cisplatin is 98%, and the binding of platinum to protein is basically irreversible [21]. The total and ultrafilterable platinum are rapidly eliminated in a biphasic manner [16], mainly via the renal pathway [22,23]. On the other hand, after a short-term intravenous infusion, the half-life of carboplatin is longer than that of cisplatin; this may be caused by the lower rate of hydrolysis, which converts carboplatin into its active form [24]. The PBR of carboplatin is 25–50%, and its ratio of PBR to irreversible PBR (which reflects the ratio of reversible protein binding) is 25% to 10%, indicating that 60–70% of its protein binding is reversible [21]. Therefore, carboplatin is less reactive toward protein, which may explain why measures of total and ultrafilterable platinum yield similar values (4337 and 3446 μg/min per mL, dosage 450 mg/m^2^) for the area under the plasma concentration-time curve (AUC), and the AUC of carboplatin is closely correlated with its clinical parameters, including toxicity and response [16,19,25,26]. The elimination of total platinum occurs in a biphasic or triphasic fashion, whereas ultrafilterable platinum is eliminated in a biphasic way. Similar to cisplatin, carboplatin is mainly excreted through the kidneys [27]. 

Oxaliplatin, another commonly used antitumor drug, binds to protein at a high ratio; moreover, it can bind to erythrocytes [11]. The maximum protein-binding rate (PBR) of oxaliplatin is 98%, and its ratio of PBR to irreversible PBR is 90% to 87% [21]. The pharmacokinetics of ultrafilterable platinum is triphasic with a short initial distribution phase and a long terminal elimination phase [28]. Similarly, it is mainly cleared through the kidneys. Nedaplatin, an analogue of cisplatin, is less reactive toward proteins in blood, and the amount of free platinum accounts for almost 50% of the total platinum. The elimination of ultrafilterable platinum is biphasic, and the pharmacokinetics of nedaplatin are generally similar to that of carboplatin [16]. Urinary excretion represents the main pathway for nedaplatin removal.

Although early studies on cisplatin’s mechanism of action suggested that DNA binding is the main reason for its antitumor activity [29,30], only 1–5% of the total intracellular platinum binds to DNA, whereas most of the platinum in blood binds to proteins, mainly by covalent binding with thiol and methionine groups [31,32]. The irreversible protein binding in blood will deactivate platinum drugs [6]. The decreased binding of cisplatin to DNA reduces its cytotoxic effect on tumor cells [33]. On the other hand, the protein binding of platinum drugs in blood plays an important role in their uptake, transport, distribution, metabolism, and excretion in vivo [34,35,36,37,38,39,40,41,42]. Irreversible binding between platinum and blood proteins can lead to toxic side effects [43]. Therefore, it is necessary to study the protein-binding behavior of platinum complexes in blood. Mass spectrometry is especially powerful in the identification and quantification of binding proteins for small-molecule drugs, including metal-based drugs [44,45,46,47,48]. Spectroscopic methods including inductively coupled plasma-atomic emission spectroscopy (ICP-AES) and atomic absorption spectrometry (AAS) can also quantitate the binding proteins of platinum drugs [49,50,51], and the cost is lower although their sensitivity and selectivity are lower than the mass spectrometric method. Thus, they are less frequently used in the relevant studies. 

## 2. Mass Spectrometry Techniques Used in Metallomics

### 2.1. Inductively Coupled Plasma Mass Spectrometry (ICP-MS)

Developed in the 1980s, ICP-MS is a method for the analysis of inorganic elements and isotopes. It combines the high-temperature ionization characteristics of inductively coupled plasma with the sensitivity and fast scanning of mass spectrometers through a unique interface. Due to its low detection limit and excellent separation power when combined with separation techniques such as liquid chromatography (LC) and capillary electrophoresis (CE), ICP-MS has become the preferred choice in metallomic analysis [52,53]. Although ICP-MS can be used for the absolute quantification of metals regardless of their morphology, it has the obvious disadvantage of molecular information being lost due to atomization of the sample in the ion source.

Size-exclusion chromatography (SEC) is usually used in LC–ICP-MS systems to separate intact proteins on the basis of their molecular weight. For example, it can be used to characterize the protein-binding properties of platinum-based and ruthenium-based drugs [54,55,56]. Recently, ICP-MS has been coupled with two-dimensional (2D) LC to achieve better performance in quantification, for example, 2DSEC–RP-LC–ICP-MS [57]. CE has the advantages of short analysis time, low sample consumption, and good compatibility with ICP-MS [58,59]; accordingly, the combination of CE and ICP-MS was used in a series of studies to explore the binding properties of metal drugs with biomolecules. For example, the CE–ICP-MS method was successfully used to identify the main binding partner of KP1019 in sera of patients participating in a phase I clinical trial [60].

### 2.2. Laser Ablation (LA)–ICP-MS

Laser ablation (LA)–ICP-MS is an imaging method for inorganic elements [61]. It relies on a laser beam to ablate the surface of analytes, which are then sent to the ICP-MS instrument via a carrier gas to obtain their elemental composition [53]. It was originally used to identify metal-containing proteins in samples separated by a gel, while it can also be applied for ultrathin tissue sections, such as kidney tissue sections of rats treated with cisplatin [62]. The main disadvantages of LA–ICP-MS are its long acquisition time and low sensitivity, which limit its use for quantification [62].

### 2.3. Electrospray Ionization (ESI)-MS

ESI-MS is widely used in proteomics studies. Complete molecular information can be obtained from complex biological samples using this method. ESI-MS can be used to determine the stoichiometric ratio of metal drugs to biomolecules, identify the binding site of metals on biomolecules through fragments, and perform large-scale proteomics analysis. Small molecules, DNA bases, and the amino acid side chains of intact proteins can all function as metal ligands. For small biomolecules, such as peptides, metal adducts can be detected on the basis of unique isotope patterns [63,64]. However, quantitative protein-binding information on drugs in blood cannot be obtained using current ESI-MS methods.

Multidimensional protein identification technology (MudPIT) is based on the combination of two-dimensional liquid chromatography (reverse-phase or strong cation exchange) and ESI tandem mass spectrometry. It is suitable for comprehensive metabolomics and proteomics analyses of biological samples in a single experiment. It has been successfully used to determine the protein-binding sites of cisplatin in human serum and of the ruthenium(II) complex in *Escherichia coli* after trypsin digestion [63,65].

## 3. Protein-Binding Behavior of Platinum Drugs in Blood Elucidated by Mass Spectrometry

Because clinically used platinum anticancer drugs are administered intravenously, their interactions with proteins in blood should be fully considered because they influence their transport, distribution, and toxicity. Some high-abundance serum proteins are responsible for the transport of small molecules, for example, human serum albumin can transport fatty acids, amino acids, and metal ions in body fluids. Various studies on the interactions of antitumor platinum complexes with plasma proteins have been performed using spectroscopic techniques, including circular dichroism (CD), fluorescence, and NMR spectroscopy [66,67,68], which provides structural information about the protein-binding behavior of platinum drugs [69]. On the other hand, the stability of platinum drugs in blood has also been studied using spectroscopic methods, such as NMR and X-ray absorption near edge structure (XANES) [70]. AAS and AES are sensitive enough to be used for quantitating binding after the removal of free drugs. However, these techniques cannot be used to analyze blood samples because of their limited selectivity. 

With the emergence of mass spectrometric and separation techniques, such as electrospray ionization mass spectrometry, inductively coupled plasma mass spectrometry, and chromatography, more detailed information about the protein-binding behavior of platinum drugs in serum can be obtained [71,72]. Extensive studies have been successfully performed to elucidate the protein-binding behavior of platinum drugs in serum, as summarized in Table 2. 

### 3.1. In Vitro Binding Analysis

In order to identify the protein-binding behavior of platinum drugs, it is necessary to evaluate the stability of platinum–protein adducts during sample preparation [46,78]. Medel et al. studied the binding of cisplatin to serum proteins using HPLC–ICP-MS and ESI-Q-TOF [73]. Pt-containing peptides were identified from the reaction mixture of cisplatin with pure transferrin (Tf) and human serum albumin (HSA). The Pt–peptide adducts were proven to be intact after tryptic digestion. Subsequently, they compared the mass spectra of platinated Tf and HSA peptide with that of the serum sample; in this way, Tf and HSA were found to bind with platinum after the incubation of human serum with cisplatin. This study provided a foundation for using a bottom-up strategy to identify the binding targets of platinum drugs. 

Thereafter, Will et al. characterized the cisplatin-binding sites in human serum proteins by combining multidimensional liquid chromatography with ESI tandem mass spectrometry [65]. Firstly, the cisplatin–serum mixture was incubated for 3 h, followed by trypsin digestion; then, peptides were separated using strong cation exchange (SCX) and reverse-phase (RP) liquid chromatography and analyzed using tandem mass spectrometry. Next, the tandem mass spectra were matched with the theoretical peptide sequence generated by the SWISS-PROT database in SEQUEST search engine. Lastly, HSA, serotransferrin (Trfe), and other abundant serum proteins (A2mg (α-2-macroglobulin), A1at (α-2-antitrypsin), Apoa1 (apolipoprotein A-I), and Apoa2) were identified. The peptide sequences and platination sites of other abundant serum proteins are listed in Table 3 [65]. To confirm their coordination sites, pure HSA and Trfe were incubated with cisplatin. All identified binding sites for HSA were confirmed using pure proteins.

To identify low-abundance binding proteins in serum, Moraleja et al. developed a shotgun method that includes peptide-based non-gel isoelectric focusing (IEF) separation [74]. Cisplatin–, oxaliplatin–, and carboplatin–protein complexes were processed using filter-aided sample preparation and trypsin digestion; then, the peptides were separated by non-gel isoelectric focusing. Since the reagents were removed after each step of the filter-aided sample preparation (FASP) method, there were no thiol-containing reagents in the focusing buffer during IEF separation; thus, the loss of platinum throughout the process was kept to a minimum. The stability of the platinum–peptide complex during FASP digestion and IEF separation was confirmed by SEC–ICP-MS. According to the ICP-MS analysis of the 24 IEF fractions, those with a higher amount of platinum were subject to subsequent nanoLC–ESI-MS/MS. The same platinum-modified peptides were found in the MS spectra of both the cisplatin–protein mixture and the human blood serum digests from the same IEF fractions, and they were assigned as platinated peptides of HSA by manually sequencing the fragmentation spectra of the species. This method has the potential to identify low abundance binding proteins, however, no binding protein was identified using the same software and parameters as that of Will et al [65]. Therefore, the search was carried out by manual inspection of the full MS spectra on the basis of the characteristic isotopic pattern of platinated peptides. 

**Figure 2:**
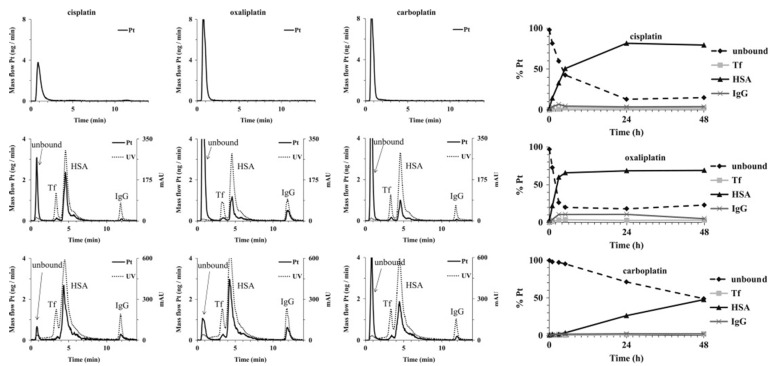
Two-dimensional separation of solutions of cisplatin, oxaliplatin, and carboplatin (20–40 ng Pt·mL^−1^) (upper row) on a conjoint LC (CLC) monolithic column followed by ICP-MS detection and 5-times diluted samples of mixtures of standard serum proteins and serum samples spiked with single Pt-based chemotherapeutics (100–200 ng Pt·mL^−1^, incubation time 24 h) (middle and lower row, respectively), followed by UV (278 nm) and ICP-MS detection (Pt mass flow is based on the measurement of isotope ratios at *m*/*z* 194 and 195). The right panel shows the interaction kinetics of cisplatin, oxaliplatin, and carboplatin with serum proteins and the distribution of Pt-based chemotherapeutics in human serum. Reprinted with permission from [79]. Copyright Elsevier 2013.

The quantification of Pt–protein adducts is necessary for assessing the dose-dependent response to treatment of individual patients. After identification of the major binding proteins in blood, quantitative analysis of the binding becomes possible. For example, a novel method was developed by Janez et al. for speciation of Pt in human serum [79]. The separation was based on affinity and ion exchange (IE) chromatographic modes using isocratic elution. A conjoint liquid chromatograph was constructed by placing one Protein G and one diethylamino (DEAE) disk in a single housing, thus enabling rapid two-dimensional separation of unbound Pt-based drugs and their complexes with proteins in human serum using a single injection. Separated Pt species were monitored online by ultraviolet (UV) and ICP-MS detection via isotope dilution (Figure 2). Compared with conventional 2D chromatographic separation method, which usually consists of SEC and IE, it was faster and simpler with satisfactory sensitivity, selectivity, method repeatability, and Pt recovery. Thus, it was subsequently used for the speciation of Pt in different samples, including the in vitro investigation of the interaction kinetics of cisplatin, carboplatin, and oxaliplatin with serum proteins and the distribution of Pt in spiked human serum (Figure 2). Kinetic studies showed that cisplatin and oxaliplatin react faster with serum proteins than carboplatin. Distribution studies showed that most of the Pt was bound with HSA, while IgG and Tf only accounted for small portions; the binding proteins were identified through a comparison with standard proteins reported as targets of platinum drug in serum. It is worth noting that these are high-abundance proteins in serum. The developed method could be useful for preclinical and clinical studies of the interaction and distribution of metallo-drugs with proteins in blood. Later, Larios et al. used a similar method to detect and accurately quantify adducts of plasma/serum proteins with carboplatin, and a similar distribution of Pt on serum proteins was obtained [80]. The reference methodology requires the usage of a Pt–HSA adduct calibrant with natural Pt isotopic composition and a ^194^Pt–HSA spike, which could be very useful for clinic analysis.

### 3.2. In Vivo Binding Analysis 

Pharmacokinetic studies of platinum drugs are mainly based on the determination of total platinum content in blood and urine. The methods most commonly used for platinum content determination are atomic absorption spectroscopy and the more sensitive ICP-MS method, which can be easily coupled with liquid chromatography. Combined with gel chromatography and reverse-phase chromatography, ICP-MS can be used to directly analyze the protein bound to Pt in plasma and detect the possible metabolites of platinum drugs. For example, Allain et al. monitored the binding of oxaliplatin to plasma proteins and the penetration of red blood cells (RBCs) using LC coupled with ICP-MS [75]. The biotransformation of oxaliplatin in plasma and urine was studied using SEC or RP-LC coupled with ICP-MS. In plasma, four platinum-containing peaks were found, and platinum was found to bind to albumin and β-globin in vivo. Inside the red blood cells, two platinum-containing peaks were found after oxaliplatin infusion, namely, hemoglobin and low molecular weight substances in RBCs.

Carboplatin is a second-generation platinum antitumor drug, commonly used to treat malignant tumors. In order to study the interactions between carboplatin and plasma proteins, Xie et al. developed an SEC–ICP-MS method to analyze the plasma of patients receiving chemotherapy, with the aim of monitoring and identifying the complexes formed between plasma proteins and carboplatin [76]. The results showed that carboplatin–albumin and carboplatin–globulin complexes were formed after carboplatin infusion, and the concentration of all platinum substances decreased as they were metabolized and continuously excreted from the human body. Furthermore, a blank plasma sample was incubated with carboplatin and analyzed using SEC–ICP-MS, with the results confirming that carboplatin formed a complex with plasma proteins, mainly albumin and gamma-globulin. To further validate the study, these two proteins were incubated with carboplatin, and then their complexes were qualitatively and quantitatively characterized. In addition to the one-to-one binding of platinum and protein, protein aggregation was also observed, and the kinetic process of carboplatin binding to albumin and γ-globulin was further studied. The initial reaction rate constant for carboplatin and albumin was determined to be 0.74 M^−1^·min^−1^, whereas that for γ-globulin was 1.01 M^−1^·min^−1^. These studies can help us to understand how carboplatin interacts with plasma proteins in human blood.

**Figure 3:**
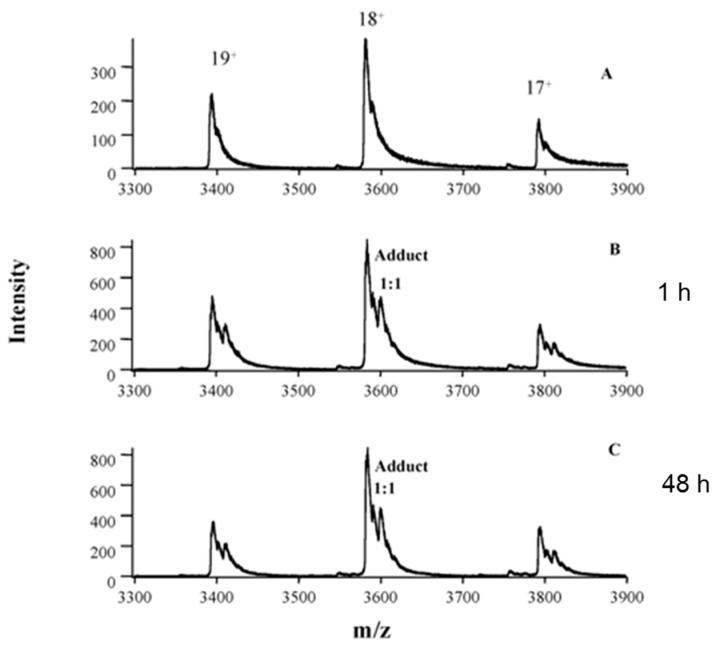
Analysis of erythrocyte samples from patient 1, who was undergoing oxaliplatin treatment, showing in vivo formation of intact Hb–oxaliplatin adducts. Spectra were obtained from erythrocytes of a healthy volunteer (**A**) and erythrocytes from blood samples of patient 1 collected at 1 (**B**) and 48 h (**C**) after the first infusion of oxaliplatin. Reprinted with permission from [43]. Copyright American Association for Clinical Chemistry 2005.

Oxaliplatin is widely used in the treatment of cancer; however, its toxicity limits its clinical application. Peng et al. used nano-electrospray tandem mass spectrometry to characterize the intact hemoglobin tetramer and its interaction with oxaliplatin [43]. The fragment ion and isotopic pattern of Hb–oxaliplatin adducts confirmed the presence of oxaliplatin. A high ratio of Hb–oxaliplatin adduct (70%) was found in the red blood cells of patients who could not tolerate oxaliplatin treatment, while a lower ratio of Hb–oxaliplatin adduct was observed in the red blood cells of another patient, who benefited from the treatment. Subsequent analysis of RBC samples from other patients further confirmed this conclusion. The blood cell samples of six patients who benefited from oxaliplatin treatment contained 25–40% adducts, whereas those of four patients with severe side-effects contained 72–82% adducts. Therefore, Hb–oxaliplatin adducts in red blood cells can be used as a clinical biomarker for evaluating toxicity and therapeutic effects. Figure 3 shows the intact Hb–oxaliplatin adducts observed in the RBC of patients receiving oxaliplatin treatment [43].

**Figure 4:**
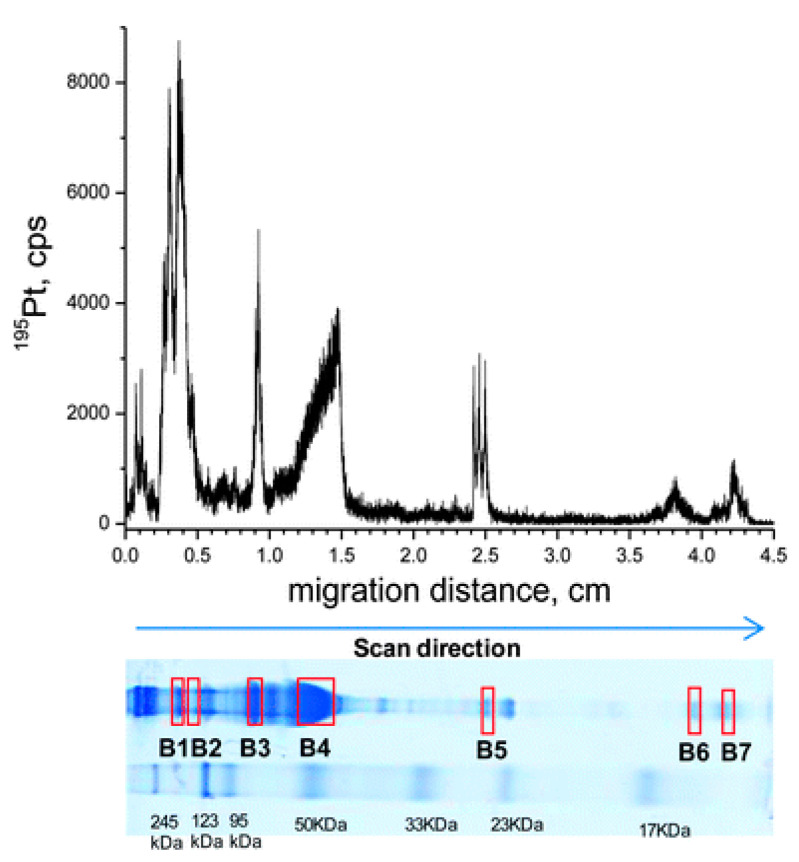
LA–ICP-MS line scan at 30 μm·s^−1^, monitoring ^195^Pt in 50 μg of serum proteins from a rat treated with 16 mg·kg^−1^ cisplatin, separated by 12.5% nrSDS-PAGE. The CBB-stained gel, dried prior to LA–ICP-MS, is displayed along with molecular weight markers. Protein bands selected for protein identification are also marked on the gel. Reprinted with permission from [77]. Copyright Royal Society of Chemistry 2012.

To identify the binding proteins of cisplatin in the serum of living animals, Gordaliza et al. developed a new method. Generally, the proteins were separated via gel electrophoresis, and the platinum-containing proteins were detected using LA–ICP-MS, followed by subsequent proteomic analysis of the band using nLC–ESI-linear ion trap quadrupole (LTQ)-Fourier transform (FT)-MS/MS [77]. Two-dimensional gel electrophoresis separation was performed under non-reducing conditions; then, Coomassie brilliant blue (CBB) or silver staining was used to show the protein bands, thus achieving a good resolution. Subsequently, LA–ICP-MS analysis was conducted to indicate the Pt-containing band, and the results showed that the platinum signal from glycerol-treated dry gels was stronger compared with that from blotted membranes. Using optimized conditions, four platinum-bound proteins, including ⍺-2-macroglobulin, transferrin, albumin, and hemoglobin, were identified in the serum of rats treated with cisplatin. Furthermore, the first complete metalloprotein profile was obtained through the use of two-dimensional gel electrophoresis with LA–ICP-MS. The CBB-stained gel is shown in Figure 4 with molecular weight markers. The bands used for protein identification are also marked on the gel with their ^195^Pt signals shown above. This method was also used to detect protein targets in cisplatin-incubated renal tubular epithelial cells. 

In another study, Kato et al. used ICP-MS to study the protein-binding properties of albumin with cisplatin, carboplatin, and oxaliplatin and measure their real-time concentration in rat plasma [21]. For in vivo binding, the maximum protein binding rates for cisplatin, carboplatin, and oxaliplatin were found to be 96%, 15%, and 80% in plasma, respectively. Therapeutic drug monitoring is often performed by measuring the real-time concentration of free drugs, whereas high protein binding interferes with the measurement. The difficulties in predicting the tissue concentrations of cisplatin and oxaliplatin from their plasma concentrations make it impossible to perform therapeutic drug monitoring. On the contrary, carboplatin binds to plasma proteins at a low ratio, thereby enabling therapeutic drug monitoring. 

### 3.3. Software for the Identification of Proteins Binding with Platinum Drugs

**Figure 5:**
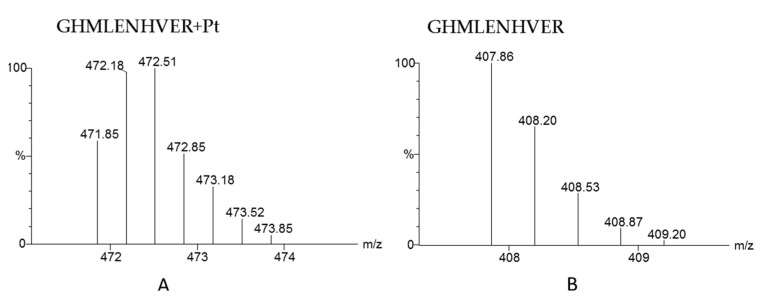
The theoretical MS spectra of platinated peptide GHMLENHVER (**A**) and the theoretical MS spectrum of peptide GHMLENHVER without Pt modification (**B**).

The protein binding with platinum anticancer drugs is usually identified by finding the peptides that are platinated. As mentioned in Section 3.1, early attempts to identify the binding proteins of platinum drugs in serum were restricted by the software for mass spectrometric data analysis. Later, various search engines were developed to identify proteins and their modifications from high-resolution mass spectrometric data, such as SEQUEST, Mascot, MaxQuant, and Pfind [81,82,83,84]. These engines are reliable and efficient in processing routine proteomic data. When used to identify proteins modified by metal drugs in complex matrixes, the software allows users add variable modifications, and platinum can be added as variable modification for database searching. For example, [Pt]^2+^, {(NH_3_)Pt}^2+^, {(NH_3_)_2_Pt}^2+^ and {(NH_3_)_2_PtCl}^+^ with additional mass of 193, 210, 227 and 263 respectively are set as variable modification of peptide for cisplatin. Considering the complexity of blood proteome, false-positive results will occur, especially those peptides with the same mass but different elemental compositions as that of platinated peptide. Thus, the proteomic search engine cannot effectively exclude false positive results. Consequently, manually checking the isotope pattern of modified peptides is the only way to exclude false-positive results; typical isotope patterns of a platinated peptide and free peptide are shown in Figure 5. As mentioned in Section 3.1, Sheldrick et al. used SEQUEST to identify the proteins bound to cisplatin in human serum; then, they made use of the characteristic isotope peaks of platinated fragment ions to manually confirm the results [65]. There is great demand for automatic software to match the isotope patterns of identified peptides with theoretical patterns.

Recently, bioinorganic chemists have made some attempts to develop data processing software. A typical example is the Apm^2^ tool developed by Dyson et al. [85,86]. It is a user-friendly webserver-based tool that can compare the experimental spectrum and the theoretical spectrum from identification results, giving a similarity ratio that can be used to automatically exclude false-positive results. Another example is the Smart Numerical Annotation Procedure (SNAP) algorithm developed by Sadler et al. [87]. This algorithm in Python code can be preprogrammed into data analysis software to assign fragments of metal-containing peptides, whose isotope pattern is different from that of ordinary peptides (Figure 5). It is worth noting that both Apm^2^ and SNAP can only be used to match and assign the binding sites of drugs on known peptides or proteins. SNAP–LC has been used to identify the binding sites of platinum, iridium, and osmium complexes on a model protein after nanoLC–MS/MS analysis [87,88,89]. The Apm^2^ tool has been utilized to locate the modification sites of cisplatin on ubiquitin using top-down CID–MS/MS [85]. 

To efficiently identify proteins modified by metal drugs in complex matrixes, the combination of an open search engine with an isotopic matching tool is necessary. Identifying the potentially modified protein is the first step, after which an automatic verification step should be conducted with the help of software such as Apm^2^ and SNAP. According to the similarity between experimental isotopes and theoretical isotopes, false-positive results can be excluded. This combination speeds up the data process for identifying proteins binding with platinum drugs in blood.

## 4. Conclusions

Platinum-based anticancer compounds are widely used for the treatment of cancer. The interactions of platinum-based drugs with proteins in the blood are vital for their uptake, distribution, metabolism, bioavailability, and toxicity. The LC–ICP-MS method was initially commonly used for the identification and quantification of binding proteins for platinum drugs in blood via comparison with standard proteins; however, the combination of multidimensional LC with ESI-MS/MS is being increasingly used. Since predominantly high-abundance proteins are identified, several attempts have been made to identify low-abundance protein targets, with no satisfactory results being achieved so far due to the restrictions of analytical methods and data processing software. Nevertheless, with the development of separation techniques and bioinformatics tools, the comprehensive characterization of binding targets for metal drugs in blood will become possible in the future. Mass-spectrometry-based analytical methods have superior selectivity and sensitivity; when combined with multidimensional separation methods and efficient software, they can play an even greater role in identifying the protein targets of metal-based drugs both in vitro and in vivo, which will be helpful to understand the pharmacokinetics and toxicity of metal-based drugs and optimize their structures.

## Figures and Tables

**Table 1:** Pharmacokinetics of cisplatin and its analogues following intravenous infusion. Reprinted with permission from [19]. Copyright Springer 2000.

	Cisplatin	Carboplatin	Oxaliplatin
t_1/2α_ (min)			
Total Platinum	14–49	12–98	26
Ultrafiltrate	9–30	8–87	21
t_1/2β_ (h)			
Total Platinum	0.7–4.6	1.3–1.7	
Ultrafiltrate	0.7–0.8	1.7–5.9	
t_1/2γ_ (h)			
Total Platinum	24–127	8.2–40	38–47
Ultrafiltrate			24–27
Protein Binding (%)	>90	24–50	85
Urinary Excretion (%)	23–50	54–82	>50

t_1/2α_, t_1/2β_, and t_1/2γ_ = early, mid, and late half-lives.

**Table 2:** Summary of the methods for studying the protein-binding behavior of platinum drugs in blood.

Drugs	Methods	Sample Type	Binding Proteins in Blood	Search Engine	Advantages	Disadvantages
Cisplatin	HPLC–ICP-MS and ESI-Q-TOF	Human serum (in vitro)	Tf, HSA [73]	-	Reliable identification	Requires pure protein; favorable for high-abundance proteins
Cisplatin	MudPIT	Human serum (in vitro)	HSA, Trfe, A2mg, A1at, Apoa1, Apoa2 [65]	SEQUEST	Platination site can be found	Unfavorable for low-abundance proteins
Cisplatin	SEC–ICP-MS and nLC–ESI-LTQ-Orbitrap-MS/MS	Human serum (in vitro)	HSA [74]	-	Reliable identification	Requires pure protein
Oxaliplatin	SEC–ICP-MS	Human plasma (in vivo)	γ-globulins, Hb, albumin [75]	-	Quantitative information for the binding can be obtained	Unfavorable for low-abundance proteins; platination site cannot be found
Carboplatin	SEC–ICP-MS	Human plasma (in vivo)	HSA, γ-globulins [76]	-	Quantitative information for the binding can be obtained	Unfavorable for low-abundance proteins; platination site cannot be found
Oxaliplatin	nanoESI-QTOF-MS/MS	Human red blood cells (in vivo)	Hb [43]	-	Semiquantitative information for the binding can be obtained	Platination site cannot be found
Cisplatin	LA–ICP-MS and nLC–ESI-LTQ-FT-MS/MS	Wistar rat serum (in vivo)	A2mg, Tf, HSA, Hb, APOC2 [77]	Mascot	Semiquantitative information for the binding can be obtained	Platination site cannot be found due to sample preparation; unfavorable for low-abundance proteins
Cisplatin, carboplatin, oxaliplatin	ICP-MS	Wistar/ST rat plasma (in vivo)	Albumin [21]	-	Quantitative information for the binding can be obtained	Platination site cannot be found

Tf, transferrin; HSA, human serum albumin; A1at, α-2-antitrypsin; Apoa1, apolipoprotein A-I; Apoa2, apolipoprotein A-II; APOC2, apolipoprotein C-II; Trfe, serotransferrin; Hb, hemoglobin; A2mg, α-2-macroglobulin.

**Table 3:** Platinated peptide sequences of other abundant proteins in blood serum. Reprinted with permission from [65].

Peptide Sequence ^[a]^	Pt Fragment Mass ^[b]^	Sample No.	Charge	SEQUEST Xcorr	Parameters ΔCn	Ions ^[c]^	Other Possible Binding Site(s) ^[d]^
1. Serotransferrin (Trfe): Residues **Y314/E385**							
(a) K·^313^M^OX^Y@LGYEYVTAIR^324^·N	210	2	2	2.86	0.10	15/22	
(b) K·^381^IM@NGEADAMSLDGGFVYIAGK^401^·C	193	1	2	4.49	0.48	22/40	**E385 (0.11)**, D387 (0.31)
(c) K·^381^IM@NGEADAMSLDGGFVYIAGK^401^·C	227	2	2	5.14	0.50	19/40	**E385 (0.18)**, D387 (0.33)
(d) M·^383^NGE@ADAM^OX^SLDGGFVYIAGK^401^·C	263	2	2	4.57	0.64	21/36	D387 (0.18)
2. α-2-Macroglobulin (A2mg): Residues **E300/S532**							
(a) K·^297^LHTE@AQIQEEGTVVELTGR^315^·Q	227	1	3	3.71	0.29	24/72	T299 (0.05), H298 (0.05)
(b) R·^517^LLIYAVLPTGDVIGDS@^532^·A	227	2	2	2.88	0.33	22/30	D531 (0.08)
3. α-1-Antitrypsin (A1at): Residues **D107/K368**							
(a) L·^104^NQPD@SQLQLTTGNGLFLSEGLK^125^·L	210	1	2	3.12	0.28	15/42	S108 (0.09)
(b) L·^04^NQPD@SQLQLTTGNGLFLSEGLK^125^·L	210	2	2	3.44	0.34	16/42	S108 (0.09)
(c) R·^102^TLNQPDS@QLQLTTGNGLFLSEGLK^125^·L	227	1	3	4.12	0.30	30/92	**D107 (0.11)**
(d) K·^366^FNK@PFVFLMIEQNTK^380^·S	193	1	3	4.67	0.16	24/56	
(e) N·^368^K@PFVFLM^ox^IEQNTK^380^	263	2	2	4.41	0.23	20/24	
4. Apolipoprotein AI (Apoa1): Residues **D48/D73/S228**							
(a) K·^46^LLD@NWDSVTSTFSK^59^·L	210	2	2	3.50	0.15	16/26	D51 (0.11)
(b) K·^60^LREQLGPVTQEFWD@N^74^·L	227	1	2	3.34	0.22	16/28	
(c) K·^227^VS@FLSALEEYT^337^·K	263	1	2	3.08	0.29	15/20	
5. Apolipoprotein AII (Apoa2): Residues **C6/E8**							
(a) P·^6^C@VESLVSQYFQTVTDYGK^23^·D	193	2	2	4.54	0.49	22/34	**E8 (0.07)**, S9 (0.08)
(b) E·^5^PC@VESLVSQYFQTVTDEYGK^23^·D	210	1	2	3.94	0.26	17/36	**E8 (0.03)**, S9 (0.14)
(c) V·^8^E@SLVSQYFQTVTDYGK^23^·D	227	1	2	5.46	0.52	22/30	S9 (0.04)
(d) P·^6^C@VESLVSQYFQYFQTVTDYGK^23^·D	263	1	2	3.65	0.25	21/34	**E8 (0.01)**, S9 (0.06)

^[a]^ The assigned binding sites in the listed platinated peptides are designated by an @ symbol following the residue. The most probable binding sites on the basis of all analyzed peptide sequences in this range are given in bold type. ^[b]^ [Pt]^2+^ = 193, {(NH_3_)Pt}^2+^ = 210, {(NH_3_)_2_Pt}^2+^ = 227, {(NH_3_)_2_PtCl}^+^= 263. ^[c]^ Ratio of assigned b^+^ and y^+^ ions to the total number of possible ions. ^[d]^ The ΔCn values of alternative neighboring sites are given in parentheses.

## Abbreviations

The following abbreviations are used in this manuscript.

A1at	α-2-antitrypsin
α2MG	α-2-macroglobulin
AES	atomic emission spectroscopy
Apoa1	Apolipoprotein A-I
Apoa2	Apolipoprotein A-II
APOC2	apolipoprotein C-II
CBB	Coomassie brilliant blue
CD	circular dichroism
CE	capillary electrophoresis
CID	collision-induced dissociation
DEAE	diethylamino
ESI-MS/MS	electrospray ionization mass spectrometry
FASP	filter-aided sample preparation
HSA	human serum albumin,
Hb	hemoglobin
HPLC–ICP-MS	high-performance liquid chromatography/inductively coupled plasma mass spectrometry
IE	ion exchange
IEF	isoelectric focusing
IgG	immunoglobulin
LA-ICP-MS	laser ablation/inductively coupled plasma mass spectrometry
MudPIT	multidimensional protein identification technology
PBR	protein-binding rate
RBC	red blood cells
SCX	strong cation exchange
SEC	size-exclusion chromatography
SNAP	Smart Numerical Annotation Procedure
Tf	transferrin
Trfe	serotransferrin
